# The prognostic advantage of preoperative intratumoral injection of OK-432 for gastric cancer patients

**DOI:** 10.1054/bjoc.2000.1599

**Published:** 2001-02

**Authors:** A Gochi, K Orita, S Fuchimoto, N Tanaka, N Ogawa

**Affiliations:** First Department of Surgery, Okayama University Medical School 2-5-1, Shikata-cho, Okayama, Japan

**Keywords:** intratumoral injection, OK-432 (picibanil), medical lymphadenectomy, preoperative immunotherapy

## Abstract

To investigate, by a multi-institutional randomized trial, the prognostic significance of the augmentation of tumour-infiltrating lymphocytes (TILs) by preoperative intratumoral injection of OK-432 (OK-432 it), a bacterial biological response modifier, in patients with gastric cancer. The 10-year survival and disease-free survival were examined and analysis of the factors showing survival benefit was performed. 370 patients who had undergone curative resection of gastric cancer were enrolled in this study and followed up for 10 years postoperatively. Patients were randomized into either an OK-432 it group or a control group. Ten Klinishe Einheit (KE) of OK-432 was endoscopically injected at 1 to 2 weeks before the operation in the OK-432 it group. Both groups received the same adjuvant chemoimmunotherapy consisting of a bolus injection of mitomycin C (0.4 mg kg^−1^i.v.) and administration of tegafur and OK-432 from postoperative day 14 up to 1 year later. Tegafur (600 mg day^−1^) was given orally and OK-432 (5 KE/2 weeks) was injected intradermally for a maintenance therapy. The TILs grades in resected tumour specimens and presence of metastasis and metastatic pattern in dissected lymph nodes were examined. Multivariate analysis was performed to determine the efficacy of OK-432 it on prognostic factors. All patients were followed up for 10 years. The overall 5- and 10-year survival rates and disease-free survival rates of the OK-432 it group were not significantly higher than those of the control group. However, OK-432 it significantly increased the 5- and 10-year survival rates of patients with stage IIIA + IIIB, moderate lymph node metastasis (pN2), and positive TILs. OK-432 it was most effective at prolonging the survival of patients who had both positive TILs and lymph node metastasis. The OK-432 it group with positive TILs showed a significant decrease in metastatic lymph node frequency and in the number of lymph node micro- metastatic foci when compared to the control group. This study showed that only one time preoperative OK-432 it, particularly when it triggers TILs, is effective for reduction of regional lymph node metastasis. OK-432 it probably acts partly by eliminating micro-metastatic foci in lymph nodes. Preoperative intratumoral injection of OK-432 is technically very easy and has no serious adverse effects, so it is a promising form of neoadjuvant immunotherapy for advanced gastric cancer. © 2001 Cancer Research Campaign http://www.bjcancer.com


British Journal of Cancer (2001) **84**(4), 443–451
© 2001 Cancer Research Campaign
doi: 10.1054/ bjoc.2000.1599, available online at http://www.idealibrary.com on IDEAL®

http://www.bjcancer.com

### The prognostic advantage of preoperative intratumoral injection of OK-432 for gastric cancer patients

**A Gochi, K Orita, S Fuchimoto, N Tanaka and N Ogawa**

First Department of Surgery, Okayama University Medical School 2-5-1, Shikata-cho, Okayama, Japan

**Summary** To investigate, by a multi-institutional randomized trial, the prognostic significance of the augmentation of tumour-infiltrating lymphocytes (TILs) by preoperative intratumoral injection of OK-432 (OK-432 it), a bacterial biological response modifier, in patients with gastric cancer. The 10-year survival and disease-free survival were examined and analysis of the factors showing survival benefit was performed. 370 patients who had undergone curative resection of gastric cancer were enrolled in this study and followed up for 10 years postoperatively. Patients were randomized into either an OK-432 it group or a control group. Ten Klinishe Einheit (KE) of OK-432 was endoscopically injected at 1 to 2 weeks before the operation in the OK-432 it group. Both groups received the same adjuvant chemoimmunotherapy consisting of a bolus injection of mitomycin C (0.4 mg $kg^{-1}$ i.v.) and administration of tegafur and OK-432 from postoperative day 14 up to 1 year later. Tegafur (600 mg $day^{-1}$) was given orally and OK-432 (5 KE/2 weeks) was injected intradermally for a maintenance therapy. The TILs grades in resected tumour specimens and presence of metastasis and metastatic pattern in dissected lymph nodes were examined. Multivariate analysis was performed to determine the efficacy of OK-432 it on prognostic factors. All patients were followed up for 10 years. The overall 5- and 10-year survival rates and disease-free survival rates of the OK-432 it group were not significantly higher than those of the control group. However, OK-432 it significantly increased the 5- and 10-year survival rates of patients with stage IIIA + IIIB, moderate lymph node metastasis (pN2), and positive TILs. OK-432 it was most effective at prolonging the survival of patients who had both positive TILs and lymph node metastasis. The OK-432 it group with positive TILs showed a significant decrease in metastatic lymph node frequency and in the number of lymph node micro-metastatic foci when compared to the control group. This study showed that only one time preoperative OK-432 it, particularly when it triggers TILs, is effective for reduction of regional lymph node metastasis. OK-432 it probably acts partly by eliminating micro-metastatic foci in lymph nodes. Preoperative intratumoral injection of OK-432 is technically very easy and has no serious adverse effects, so it is a promising form of neoadjuvant immunotherapy for advanced gastric cancer. © 2001 Cancer Research Campaign http://www.bjcancer.com

**Keywords**: intratumoral injection; OK-432 (picibanil); medical lymphadenectomy; preoperative immunotherapy

Previous research has indicated that cancer patients with high grades of tumour-infiltrating lymphocytes (TILs) have a better prognosis for many tumours, including gastric cancer (MacCarty and Mahle 1921, Black et al, 1956; Miwa et al, 1984).

A number of recent studies have also demonstrated that tumour-specific cytotoxic T cells (CTLs) are present in TILs from patients with tumours of various types, including melanoma (Itoh et al, 1986; Topalian et al, 1989), renal cancer (Schenidel et al, 1993), ovarian cancer (Ionaaides et al, 1993), sarcoma (Slovin et al, 1986), squamous cell cancer of the head and neck (Yasumura et al, 1994), and gastric cancer (Ikeda et al, 1996). About 35 years ago, we found an increase of TILs in resected gastric cancer specimens after intratumoral injection of BCG 1 to 2 weeks before operation. Subsequently, we have performed preoperative intratumoral injection of various biological response modifiers (BRMs), such as BCG-CWS, Nocardia rubra-CWS, OK-432, PSK, and IL-2, in gastric cancer patients (Miwa et al, 1984; Moriyasu et al, 1985; Akura et al, 1986). Any of these BRMs injected 1 to 2 weeks before operation trigger an increase of TILs in the resected tumours of two-thirds of the patients. Preliminary study on preoperative intratumoral injection of OK-432 (OK-432 it) was performed, because OK-432 has been a commercially available BRM which has been widely used in Japan for over 20 years. TILs were moderately positive in 40% and markedly positive in 33% of the patients with preoperative OK-432 it compared with only 31% and 0%, respectively, in the control group without OK-432 it (Miwa et al, 1984). It remains of great interest to determine whether TILs induced by OK-432 it can improve the postoperative prognosis of gastric cancer patients.

Although there has been remarkable development of gastroscopy for the early detection of gastric cancer as well as improvement of surgical techniques during the last 3 decades, the 5-year survival rate of advanced gastric cancer patients undergoing curative resection has remained at 20 to 49% for stage III and 5 to 15% for stage IV (The Japanese Gastric Cancer Study Group 1981; Siewert et al, 1993). Nevertheless, the majority of such patients die of a recurrence based on microscopic residual tumours. In order to eradicate those microscopical residual tumours postoperative adjuvant chemotherapy with intravenous mitomycin C (MMC) and oral 5-FU or 5-FU derivatives like tegafur has been routinely performed for advanced gastric cancer patients in Japan for the last 20 years (Inokuchi et al, 1984; Maehara et al, 1991; Nakazato, 1994). Subsequently, systemic nonspecific immunotherapy with OK-432 has been widely combined with adjuvant chemotherapy, because immunotherapy is generally more effective when the residual tumour mass is quite small (Arinaga et al, 1992).

*Received 21 December 1999*
*Revised 25 July 2000*
*Accepted 1 November 2000*

*Correspondence to:* A Gochi

On the basis of such adjuvant chemoimmunotherapy for gastric cancer patients, we conducted a multi-institutional randomized control trial of adjuvant chemoimmunotherapy consisting of MMC, tegafur, intradermal injection of OK-432 with or without preoperative OK-432 it in gastric cancer patients between June 1985 and December 1986.

The present article is the final report. According to the UICC: TNM classification of gastric cancers (4th edition), we found that OK-432 it significantly improved the 10-year survival of gastric cancer patients with stage IIIA+IIIB, positive TILs, $pN_2$ lymph node metastasis. In particular, OK-432 it was useful for patients with positive TILs and pN(+) when used in combination with adjuvant chemoimmunotherapy.

### MATERIALS AND METHODS

### Stratification and randomization of patients

The procedures have been described previously (Tanaka et al, 1994). Briefly, from April 1985 to December 1986, patients with gastric cancer determined endoscopically to be advanced but curatively resectable were randomly enrolled into either the OK- 432 it group or the control group after kindly informed consent.

### Protocol and postoperative treatment

Patients in the OK-432 it group had endoscopic injection of OK-432 (10 KE 5 $ml^{-1}$ saline) into approximately 10 sites in the tumour using NM-1K needles (Olympus Co, Japan) at 7 to 14 days before surgery. Patients in the control group were not treated before the operation. All patients received identical postoperative chemoimmunotherapy. MMC (0.4 mg $kg^{-1}$) was injected intravenously immediately after the operation. Oral tegafur (600 mg $day^{-1}$) and intradermal injection of OK-432 (5 KE 2 $weeks^{-1}$) were given from postoperative day 14 for up to 1 year later as maintenance therapy.

### OK-432

OK-432 is a penicillin-treated lyophilized powder of the avirulent Su strain of human Group A *Streptococcus pyogenes* (Chugai Pharmaceutical, Tokyo, Japan). The Klinische Einheit (KE) unit is used to express the strength of the preparation, with 1 KE of OK-432 being equivalent to 0.1 mg of dried streptococcal cells.

### Tegafur

Tegafur (1-(2-tetra hydrouranyl)-5-fluorouracil, MW:200) is a prodrug that is slowly metabolized to 5-FU by microsomal cytochrome p450, which is mainly found in the liver, and soluble enzymes. Tegafur has comparable activity with that of 5-FU.

### Histopathological examination

To assess treatment efficacy, haematoxylin-eosin stained resected specimens of the main tumour and dissected lymph nodes were examined independently by 3 pathologists, and the mode evaluations was determined.

TILs were graded as absent, mild, moderate, or marked, with absent or mild TILs being classified as negative and moderate or marked TILs as positive. A total of 2829 dissected lymph nodes was examined for metastasis. The pattern of nodal metastasis was classified as minor-solitary, minor-multiple, major-solitary and major-multiple. The classifications of ‘minor’ or ‘major’ was based on the size of metastatic foci, i.e., under or over 0.2 mm in diameter, while ‘solitary’ or ‘multiple’ was based on the number of metastatic foci on each node.

### Statistical analysis

The chi-squared test was used to evaluate the clinical characteristics. The survival rate and disease-free survival rate were estimated by the Kaplan-Meier method, and statistical significance was evaluated by the log-rank test. The comparison of histological findings was performed with Student’s *t*-test. Cox’s proportional hazards regression was used to assess the impact of treatment after adjustment for other characteristics. Statistical analysis, including multivariate analysis, was performed with Statistical Analysis System (SAS) software.

### RESULTS

### Clinical and pathological characteristics

Table 1 compares the two groups with respect to sex, age, macroscopic stage, lymph node metastasis, lymph node dissection, operative curability and microscopic findings due to postoperative evaluation. Some cases with distant metastasis were enrolled for difficulty of pre-operative staging due to micro foci of liver or peritoneum. Excluding age, there were no significant differences between the two groups, and the age difference had no significant influence on survival according to multivariate analysis and adjusted analysis by age. These were no differences in background between the 39 patients in the OK-432 it group and the 32 patients in the control group with stage IIIA+IIIB.

The grade of TILs in the OK-432 it group were 4 cases in absent, 104 cases in mild, and 51 cases in moderate or marked and in the control group, 13, 100 and 38, respectively. There was a significant difference in the grade of TILs between two groups ($P = 0.038$).

There were no significant differences between two arms in the total doses of adjuvant chemoimmunotherapy. 55% of OK-432 it group and 58% of control group were administrated more than 75% of the planned doses.

### Survival and disease-free survival

At the end of December 1996, all subjects had been followed up for 10 years. 187 of the 370 eligible patients had died. 93 out of 187 patients died of cancer or other disease in the OK-432 it group and 94 out of 183 died in the control group. The survival curves for the two groups are shown in Figure 1A. The overall 5-year survival rate was 58.7% for the OK-432 it group and 56.8% for the control group, while the 10-year survival rate was 49.5% and 48.2%, respectively ($P = 0.5085$). The disease-free survival rate of curatively resected patients was also not different for the two groups throughout the follow-up period ($P = 0.2005$) (Figure 1B).

© 2001 Cancer Research Campaign

**Table 1** Clinical and pathologic characteristics

| Characteristics | Enrolled all patients (n = 370) | | | Histological stage IIIA+IIIB (n = 71) | | |
|---|---|---|---|---|---|---|
| | OK-432 it (*n* = 187) | Control (*n* = 183) | Chi square | OK-432 it (*n* = 39) | Control (*n* = 32) | Chi square |
| Sex | | | 0.002 | | | 0.330 |
| Male | 123 | 120 | NS | 23 | 21 | NS |
| Female | 64 | 63 | | 16 | 11 | |
| Age (yrs) | | | 15.996 | | | 2.837 |
| Up to 39 | 11 | 1 | $P < 0.01$ | 2 | 0 | NS |
| 40–49 | 27 | 19 | | 3 | 5 | |
| 50–59 | 61 | 48 | | 14 | 10 | |
| 60–69 | 63 | 74 | | 15 | 12 | |
| Over 70 | 25 | 41 | | 5 | 5 | |
| Depth of invasion (histological) | | | 2.579 | | | 1.886 |
| pT1 | 42 | 51 | NS | 0 | 0 | NS |
| pT2 | 88 | 78 | | 11 | 13 | |
| pT3 | 40 | 38 | | 27 | 19 | |
| pT4 | 14 | 11 | | 1 | 0 | |
| Unknown | 3 | 5 | | 0 | 0 | |
| Nodal involvement (histological) | | | 4.120 | | | 1.525 |
| pN0 | 76 | 79 | NS | 1 | 0 | NS |
| pN1 | 67 | 49 | | 18 | 12 | |
| pN2 | 27 | 29 | | 20 | 20 | |
| pM1 | 11 | 17 | | | | |
| Unknown | 6 | 9 | | | | |
| Distant metastasis | | | | | | 0.484 |
| M0 | 157 | 150 | | 39 | 32 | NS |
| M1 | 29 | 32 | | | | |
| Pathological stage | | | 1.921 | | | 0.01 |
| IA | 35 | 40 | NS | – | – | NS |
| IB | 39 | 38 | | | | |
| II | 38 | 33 | | | | |
| IIIA | 30 | 25 | | 30 | 25 | |
| IIIB | 9 | 7 | | 9 | 7 | |
| IV | 31 | 37 | | | | |
| Unknown | 6 | 8 | | | | |
| Operation method | | | 1.715 | | | 0.104 |
| Total | 42 | 40 | NS | 12 | 11 | NS |
| Extended gastrectomy | 141 | 136 | | 27 | 21 | |
| Non-resectable | 4 | 7 | | 0 | 0 | |

NS: not significant. According to the UICC classification (4th edition).

### Incidence and site of recurrence

The sites of recurrence were identified during follow-up in 71 of the 370 patients who underwent curative resection. All types of recurrence were reduced in the OK-432 it group compared with the control group and both regional lymph nodal and haematogeneous recurrence were markedly reduced (Table 2).

### Effect of stage, TILs grade, and extent of lymph node metastasis

OK-432 it significantly improved the survival rate of stage IIIA+IIIB patients (Figure 2). Among the patients who were negative for TILs, there was no difference in survival between the OK-432 it and the control groups. However, among TILs-positive patients, the OK-432 it group showed a 30% higher survival rate than the control group from the 2nd year after surgery ($P$ = 0.0149). The survival rate of patients with pN2 was also higher in the OK-432 it group than in the control group ($P$ = 0.0552) (Figure 3).

### Relationship among survival rate, TILs grade, and extent of lymph node metastasis (Figure 4)

Among TILs-positive patients who had lymph node metastasis, OK-432 it was found to be most effective in improving the survival rate ($P < 0.001$).

### Relationship between TILs grade and the frequency of metastatic lymph nodes

There was no difference between the OK-432 it group and the control group in the frequency of patients having metastatic lymph nodes.

Although, the number of dissected lymph nodes per patient was similar in both groups, irrespective of the presence or absence of OK-432 it or TILs. However, a significant difference was found in the number of metastatic lymph nodes per patient. The ratio of metastatic nodes were markedly lower in the OK-432 it group with positive TILs than in the control group with positive TILs (Table 3). Only in the OK-432 it patients with positive TILs did the number of metastatic lymph nodes per patient decrease

© 2001 Cancer Research Campaign 

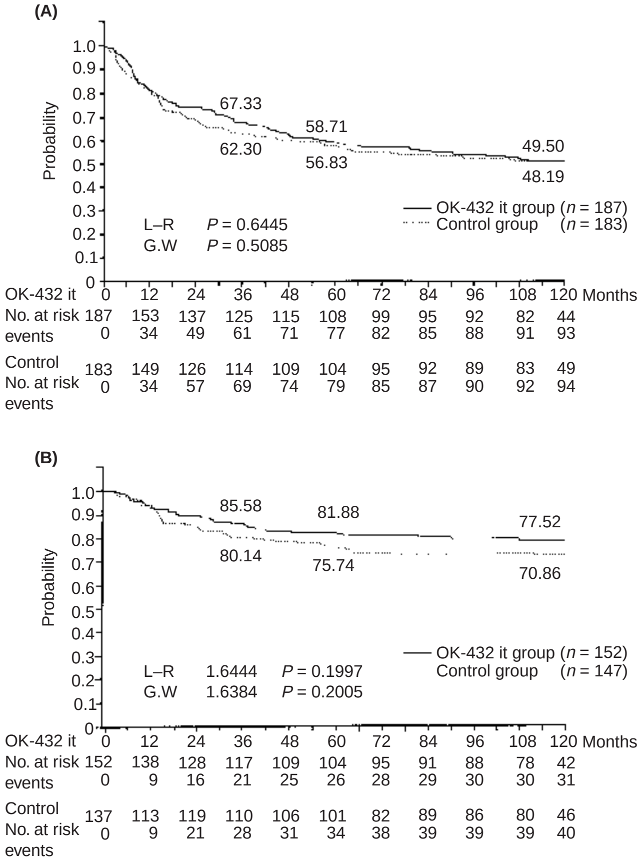

**Figure 1**(**A**) Overall survival curves for gastric cancer patients with and without preoperative intratumoral injection of OK-432. (**B**) Disease-free survival curves for gastric cancer patients with and without preoperative intratumoral injection of OK-432

significantly in the regional through the distant node groups. The number of metastatic lymph nodes per patient in the regional node group was 2.11 in the OK-432 it patients with positive TILs, 4.47 in the OK-432 it patients with negative TILs, 4.62 in the control patients with TILs, and 4.72 in the control patients without TILs.

### Relationship between OK-432 it and pattern of lymph node metastasis (Table 4)

The minor metastatic foci was smaller in the OK-432 it group than in the control group, particularly in the distant nodes from the main tumour than in the regional (perigastric and periarterial) nodes, suggesting that minor metastatic foci may be eliminated by OK-432 it.

### Multivariate analysis of prognostic factors (Table 5)

8 variable factors, which were the extension of the primary tumour (pT), the extent of regional lymph node metastasis (pN), sex, age, venous invasion, lymphatic invasion and TILs, were selected by Cox's regression analysis. In the control group, pN was the most important prognostic factor. On the other hand, in the OK-432 it group, TILs was a significant factor rather than pN.

**Table 2** Incidence and site of recurrence of curatively resected cases

| | OK-432 i.t (%) (*n* = 152) | Control (%) (*n* = 147) |
|---|---|---|
| **No. of recurrent cases** | **31 (20.4)** | **40 (27.2)** |
| Recurrence site | | |
| Peritoneum | 20 (13.2) | 22 (15.0) |
| Local | 2 | 3 |
| Vesseral type* | 15 | 27 |
| Lymph node | 4 (3.6) | 9 (9.3) |
| Haematogenic | 11 (9.8) | 18 (12.2) |
| Liver | 6 | 10 |
| Lung | 1 | 4 |
| Bone | 4 | 3 |
| Brain | 0 | 1 |

Included multi-focal recurrence sites. *$P = 0.034$ by $\chi^2$ test control vs. OK – 432 it.

### DISCUSSION

The mortality rate from gastric cancer has been decreasing worldwide (Howson et al, 1986; Stat Bite, 1998), however, gastric cancer remains one of the major causes of death in Japan. Despite the progress of endoscopic diagnosis and surgical procedures in the last 3 decades, the survival rate after curative resection of advanced gastric cancer has remained low (The Japanese Gastric Cancer Study Group, 1981; Siewert et al, 1993). There has been and still is no established adjuvant chemotherapy for gastric cancer. Accordingly, various type of adjuvant chemotherapy and/or adjuvant chemoimmunotherapy have been performed to improve the survival rate after operation for advanced gastric cancer. In those days of protocol planning, administration of intravenous MMC and oral tegafur (fluorouracil) had been the standard adjuvant chemotherapy for postoperative gastric cancer patients in Japan. Tegafur (Grem, 1996) is orally administered as a prodrug of 5-FU. Phase II trials in various solid tumours have indicated that tegafur has an activity equal to that of 5-FU. Adjuvant chemoimmunotherapy has been extensively performed by systemic administration of OK-432 for a variety of malignancies (Watanabe and lwa, 1984; Cervical Cancer Immunotherapy Group, 1987; Arinaga et al, 1997). OK-432 is an immunomodulatory agent prepared from an avirulent human strain of *Streptococcus pyogenes*. It has been shown to induce various cytokines, especially Th1 cytokines (Saito et al, 1982; Yamamoto et al, 1986; Yang et al, 1992) and to upregulate both perforin (Kataoka et al, 1991) and Fas ligand expression (Toda et al, 1996; Sato et al, 1997) of immune cells. In accordance with these findings, OK-432 is also known to enhance or activate the cytotoxic activity of various effector cells (Ishii et al, 1976; Uchida and Micksha, 1983), which may be responsible for its therapeutic effect. The augmentation of perforin (Kataoka et al, 1991), induction of IL-2 and IFN-γ, and the upregulation of Fas ligand (Toda et al, 1996; Sato et al, 1997) by OK-432 may play a major role in promoting anti-tumour necrotic and apoptotic effects of immune cells like NK cells, LAK cells, CTL (Kagii et al, 1994; Lowin et al, 1994; Lin et al, 1995; Frost et al, 1997), and TILs. These such biological activity of OK-432 are thought to occur at the tumour site in the case of intratumoral injection of OK-432. In our previous studies (Gotoh et al, 1991). TILs induced by OK-432

© 2001 Cancer Research Campaign

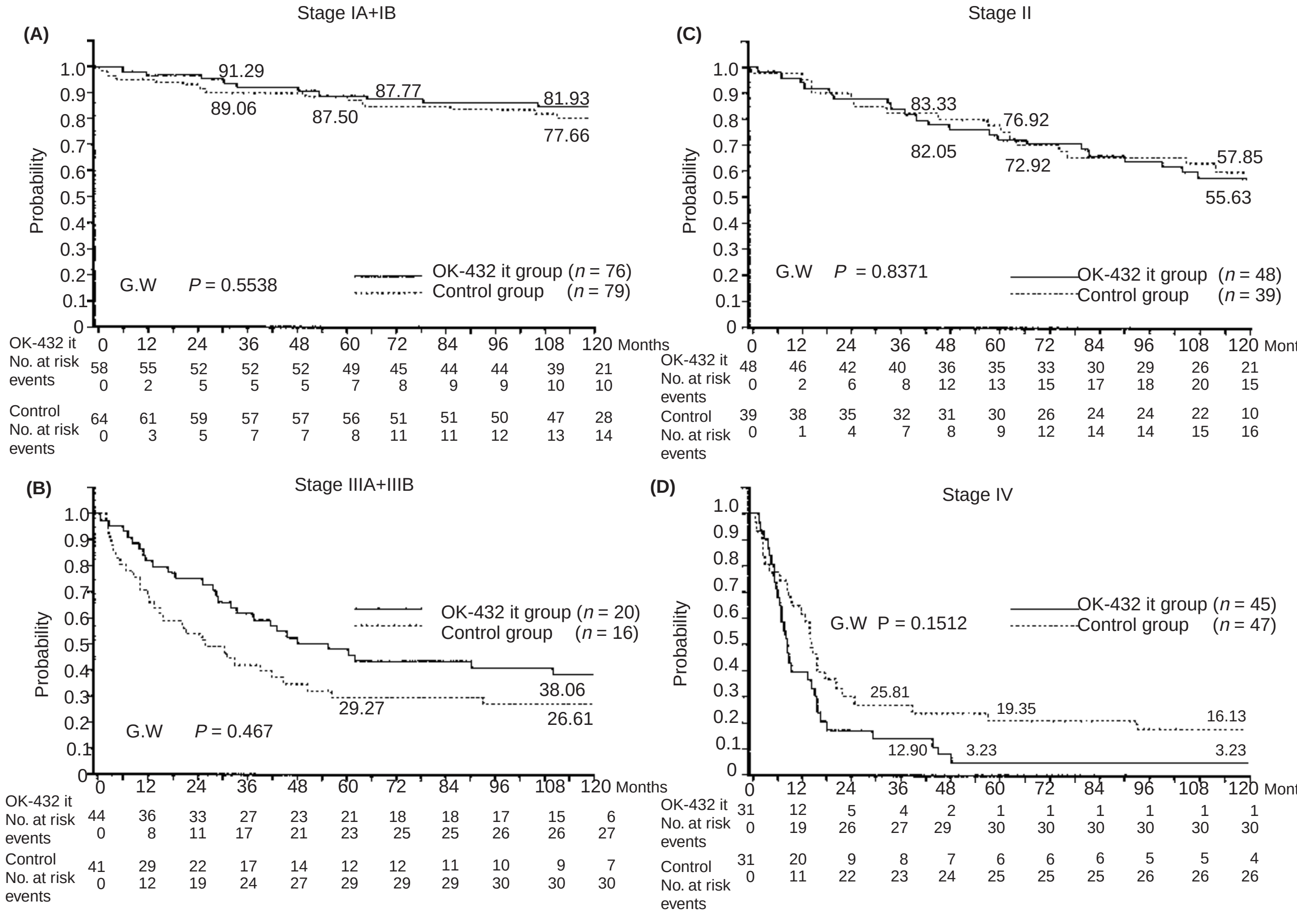

**Figure 2** Survival curves for gastric cancer patients with and without preoperative intratumoral injection of OK-432 according to clinical stage it showed augmented NK- and LAK activity, whereas TILs from patients without OK-432 it had no antitumor activity. Considerable destruction of tumour cells was also found in tumour specimens from patients with OK-432 it. Recently, Khalili et al (1998) reported that in ovarian cancer patients the spontaneous production of IL-10 and TNF-α were higher in TILs than in peripheral blood mononuclear cells, and the anti-CD3-induced IL-12 production was dramatically lower in TILs. These biased Th2-dominant cytokines might contribute to the anergic T cell function against tumour cells in TILs. IL-10 is also produced by various human solid tumours and could serve to blunt a natural or induced antitumor immune response in the tumour site (Maguri et al, 1997). Lopez et al (1998) also reported that TILs from breast cancer were defective both in proliferate response and IL-2 receptor mRNA expression but recovered when incubated in the presence of recombinant IL-2. These findings support that anergic TILs in gastric cancer patients may recover the antitumor activity in the presence of Th1-dominant cytokines including IL-2 and IFN-γ induced by OK-432 it. When dissected regional lymph nodes from OK-432 it patients were stained with an anti-OK-432 antibody, macrophages containing OK-432 particles were found in all the dissected nodes from perigastric to para-aortic (data not shown). We previously reported that lymph nodes and TILs from patients with OK-432 it also demonstrated NK- and LAK activity, but no antitumor activity was seen in the nodes from patients without OK-432 it (Gotoh K et al, 1991). In this study, we reconfirmed that the grade of TILs was significantly increased in the OK-432 it group. The antitumor activity of TILs and lymph nodes activated by OK-432 it may be responsible for improving the 10-year survival rate of patients with stage IIIA+IIIB with positive TILs, and with both positive TILs and positive node metastasis in the group of OK-432 it. Our follow-up study of recurrence showed that regional lymph node metastasis and haematogeneous metastasis were significantly lower in the group with OK-432 it. The number of metastatic lymph nodes was markedly decreased in the OK-432 it patients with positive TILs. In our multivariate analysis of 8 prognostic factors, pN was the most important prognostic factor in the control group but it was not significant in the OK-432 it group. The number of dissected lymph nodes with micrometastasis was decreased in the OK-432 it group. Moreover, the number of metastatic lymph nodes per patient was significantly decreased in the OK-432 it patients with positive TILs. These findings suggest the possibility of elimination of micrometastatic foci in regional lymph nodes by OK-432 it. Because we previously reported that injected OK-432 distributed in the regional lymph nodes, lymphocytes of TILs or regional lymph nodes co-cultured with OK-432 produced TNF-α IFN-γ and other cytokines within 4 to 12 hours and obtained cytotoxicity for tumour cells. And also multivariated analysis suggests that the younger patients who have not so impaired immune responsibility were more suitable for this therapy.

© 2001 Cancer Research Campaign 

**Table 3** Relationship between grade of TILs and grade of lymph node metastasis

| TI Ls | Metastatic incidence by case (%) Metastatic cases/Whole cases OK-432 i.t. | Control | Metastatic incidence by node (%) Metastatic nodes/Whole nodes OK-432 i.t. | Control |
|---|---|---|---|---|
| No–mild | 57.5 | 57.7 | 21.7 | 21.0 |
| Moderate–marked | 58.8 | 56.8 | *9.4* | 22.9 |

OK-432 i.t. vs control of metastatic incidence by node: $P < 0.001$.

**Table 4** Morphologic pattern of metastatic foci on lymph nodes

| | OK-432 i.t. | | | | Control | | | |
|---|---|---|---|---|---|---|---|---|
| | Minor | | Major | | Minor | | Major | |
| | Solitary | Multiple | Solitary | Multiple | Solitary | Multiple | Solitary | Multiple |
| Regional | | | | | | | | |
| Perigastric | 67 | 10 | 46 | 141 | 103 | 11 | 43 | 158 |
| Periarterial | 20 | 2 | 12 | 39 | 24 | 4 | 21 | 29 |
| Distal | | | | | | | | |
| Para-aortic | 1 | 0 | 4 | 14 | 3 | 0 | 0 | 5 |
| Total | 88 | 12 | 62 | 194 | 130 | 15 | 64 | 192 |

The ratio of minor type in OK-432 is significantly reduced than that in control ($P = 0.018$).

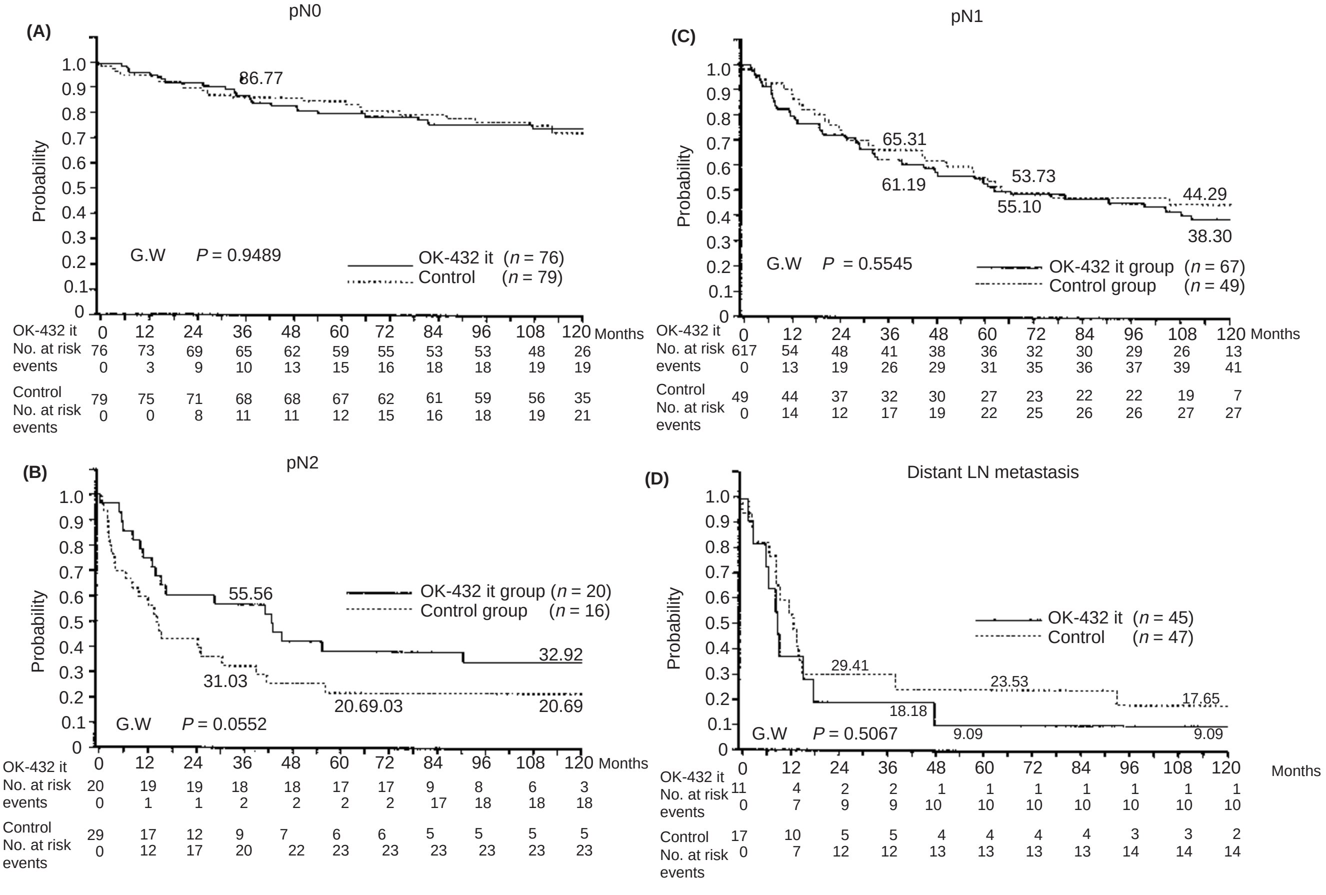

**Figure 3** Relationship between grade of extent of lymph node metastasis and survival rate for gastric cancer patients with and without preoperative intratumoral injection of OK-432

**Table 5** Multivariate analysis for survival risk

| | Control | | OK-432 it | |
|---|---|---|---|---|
| Variables § | Wald Chi-Square | *P* value | Wald Chi-Square | *P* value |
| pN | 4.4967 (1.335: 1.022–1.743) | 0.034 | 1.2152 (1.228:0.852–1.779) | 0,270 |
| pT | 4.0119 (1.431: 1.008–2.032) | 0.045 | 5.0619 (1.683: 1.069–2.648) | 0.025 |
| v | 3.5587 (1.289:0.990–1.679) | 0.059 | 0.0126 (0.980: 0.694–1.386) | 0.911 |
| ly | 1.7196 (1.209: 0.910–1.606) | 0.189 | 4.0778 (1.363: 1.0.09–1.841) | 0.044 |
| INF | 0.8629 (1.235: 0.791–1.928) | 0.353 | 0.0628 (0.947:0.621–1.445) | 0.802 |
| TIL | 0.2830 (1.181: 0.640–2.178) | 0.595 | 11.083 (0.329: 0.171–0.633) | 0.001 |
| sex | 0.2761 (0.871: 0.520–1.459) | 0.599 | 0.003 (0.983: 0.557–1.735) | 0.953 |
| age | 0.0030 (0.992: 0.738–1.333) | 0.956 | 5.196 (1.347: 1.043–1.739) | 0.023 |

v: the degree of venous invasion; ly: the degree of lymphatic invasion; INF: the pattern of infiltrating growth into the surrounding tissue. These classifications were according to Japanese Classification of Gastric Carcinoma (First English Edition).

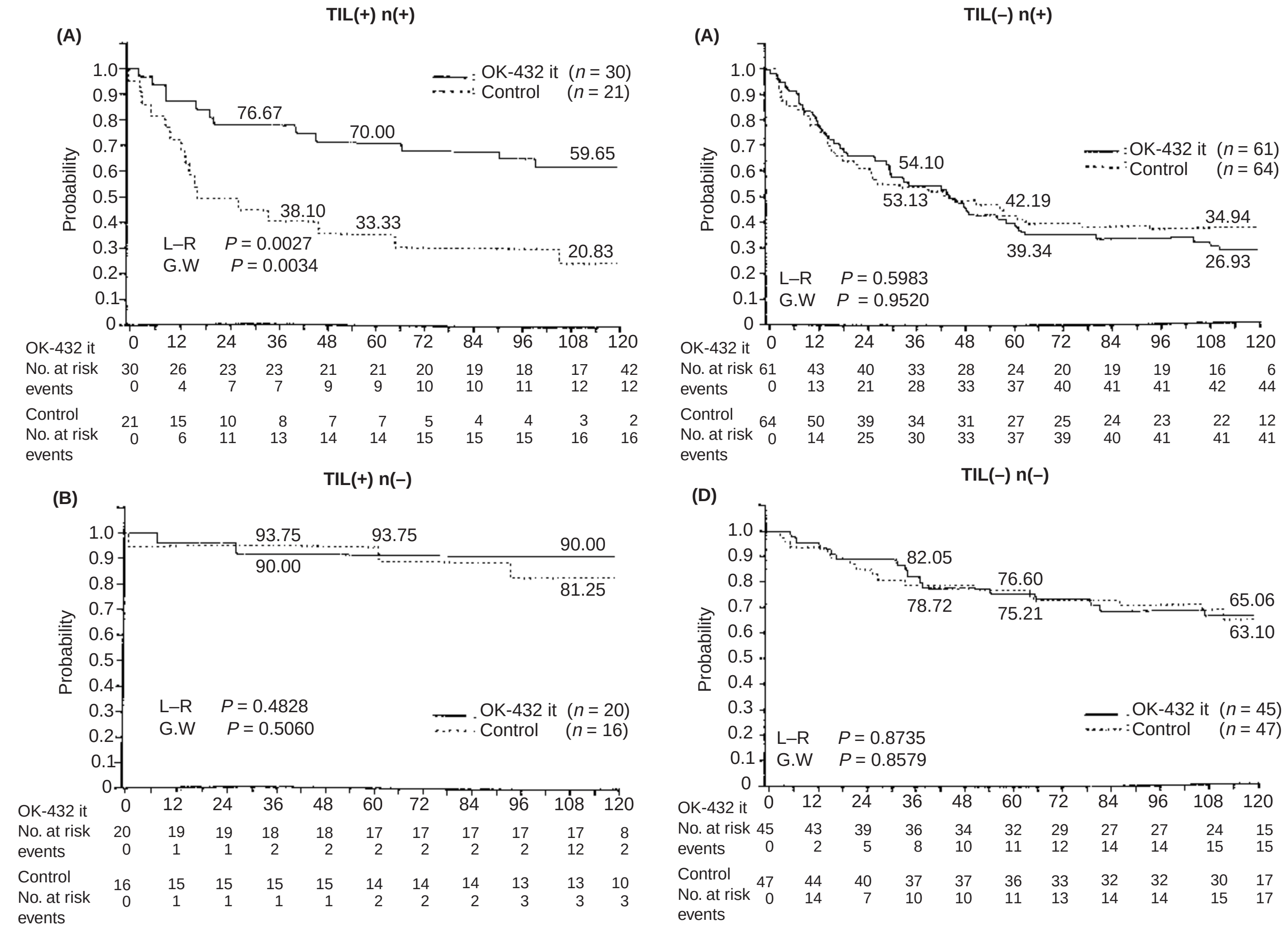

**Figure 4** Relationship among survival rate, TILs grade, and extent of lymph node metastasis

Advanced gastric cancer patients who are positive for TILs after OK-432 it should be considered as responders to OK-432, to whom adjuvant immunotherapy with intradermal injection of OK-432 should be continued in order to suppress recurrence over the long term, particularly in stage IIIA+IIIB.

The eradication of micro-metastasis is a most important strategy to improve the prognosis of cancer patients. Our study shows that preoperative intratumoral injection of OK-432 will be a more promising neoadjuvant immunotherapy for advanced gastric cancer patients.

### ACKNOWLEDGEMENT

We would like to thank the members of Chugai Pharmaceutical Co. for their perfect follow up and co-ordination in this trial.

### REFERENCES

Akura Y, Tanaka N, Kobayashi G, Gotch K, Gouchi A, Kamitani S, Gangi J and Orita K (1986) The effects of intratumoral OK-432 injection in gastric cancer patients. In: *New Application of OK-432*. Tobe T (ed) pp. 75–86 Excepta Medica: Tokyo

Arinaga S, Karimine N, Takamuku K, Nanbara S, Inoue H, Abe R, Watanabe D, Matsuoka H, Ueo H1 and Akiyoshi T (1992) A trial of adjuvant chemoimmunotherapy with mitomycin C and OK-432 for stage III gastric carcinoma. *J Surg Oncol* **50**: 187–189

Black MM, Opler RS and Speer DF (1956) Structural representations of tumor–host relationships in gastric carcinoma. *Surg Gynec Obstet* **102**: 599–603

Cervical Cancer Immunotherapy Group (1987) Immunotherapy using the streptococcal preparation OK-432 for the treatment of uterine cervical cancer. *Cancer* **60**: 2349–2402

Frost P, Ng CP, Belldegrun A and Bonavida B (1997) Immunosensitization of prostate carcinoma cell lines for lymphocytes (CTL, TIL, LAF)-mediated apoptosis via the Fas-Fas-ligand pathway of cytotoxicity. *Cell Immunol* **180**: 70–83

Gotoh K, Gouchi A, Akura Y, Tanaka N and Orita K (1991) Augmentation of cytotoxicity of tumor infiltrating lymphocytes by biological response modifiers. *Int J Immunopharmacol* **13**: 485–492

Grem JL (1996) 5-Fluoropyrimidines. In: *Cancer Chemotherapy and Biotherapy: Principles and Practice*. Chabner BA, Longo BL (eds) pp. 149–211 Lippincott-Raven: Philadelphia, New York

Howson CP, Hiyama T and Wynder EL (1985) The decline in gastric cancer: Epidemiology of an unplanned triumph. Epidemiol Rev **8**: 1–27, 1986

Ichimura O, Suzuki S, Saito M, Saito M, Sugawara Y, and Ishida N: Augmentation of interleukin 1 and interleukin 2 production by OK-432. *Int J Immunopharmacol* **7**: 263–270

Ikeda H, Sato N, Matsuura A, Sasaki A, Takahashi S, Kozutsumi D, Kobata T, Okumura K, Wada Y, Hirata K and Kikuchi K (1996) Clonal dominance of human autologous cytotoxic T lymphocytes against gastric carcinoma: Molecular stability of CDR3 structure of T cell receptor αβ gene. *Int Immunol* **8**: 75–82

Inokuchi K, Hattori T, Taguchi T, Abe O and Ogawa N(1984) Postoperative adjuvant chemotherapy of gastric carcinoma. Analysis of data on 1805 patients followed for 5 years. *Cancer* **53**: 2393–2397

Ioannides CG, Fisk B, Jerome KR, Irimura T, Wharton JT and Finn OJ (1993) Cytotoxic T cells from ovarian malignant tumors can recognize polymorphic epithelial mucin core peptides. *J Immunol* **151**: 3693–3703

Ishii Y, Yamaoka H, Toh K, and Kikuchi K (1976) Inhibition of tumor growth in vivo and in vitro by macrophages from rats treated with a streptococcal preparation, OK-432. *Jpn J Cancer Res* **67**: 115–119

Itoh K, Tilden AB and Balch CM (1986) Interleukin-2 activation of cytotoxic T-lymphocytes infiltrating into human metastatic melanoma. *Cancer Res* **46**: 3011–3017

Kagi D, Vignaux F, Ledermann B, Depraetere V, Nagata S, Hengartner H and Golstein P (1994) Fas and perforin pathway as major mechanisms of T-cell mediated cytotoxicity. *Science* **265**: 528–530

Kataoka K, Naomoto Y and Orita K (1991) In vitro induction of perforin mRNA and cytotoxic activities in human splenocytes by streptococcal preparation, OK-432. *Jpn J Clin Oncol* **21**: 330–333

Khalili H, Gal and D, Menzin AW (1998) The oligoclonality and ctyokine secretion in TIL and PBMC of patients with ovarian cancer. *FASEB J* **12**: A890, (abstr)

Lin CC, Walsh CM and Young JD (1995) Perforin: structure and function. *Immunol Today* **16**: 194–201

Lopez C, Feiner H, Rao TD, Shapiro R, Marks JR and Frey AB (1998) Repression of IL-2 mRNA translation in primary human breast cancer tumor-infiltrating lymphocytes. *Cell Immunol* **190**: 141–155

Lowin B, Hahne M, Mattmann C and Tschopp J (1994) Cytolytic T-cell ghjjkcytotoxicity in mediated through perforin and Fas lytic pathways. *Nature* **370**: 650–652

MacCarty WC and Mahle AE (1921) Relation of differentiation and lymphocytic infiltration to postoperation longevity in gastric carcinoma. *J Lab Clin Med* **6**: 473–480

Maehara Y, Moriguchi S and Sakaguchi Y (1991) Adjuvant chemotherapy enhances long-term survival of patients with advanced gastric cancer following curative resection. *J Surg Oncol* **45**: 169–172

Maguri HC Jr, Ketcha KA and Lattime EC (1997) Neutralizing anti-IL-10 antibody upregulates the induction and elicitation of contact hypersensitivity. *J Interferon Cytokine Res* **17**: 763–768

Miwa H, Gohchi A, Moriyasu F and Orita K (1984) Increase in T cell infiltration in carcinoma tissue and regional lymph node reaction to gastric cancer after local injection of immunomodulators before gastrectomy. In: *Manipulation of Host Defense Mechanisms*. Aoki T, Tsubura E, Urushizaki I (eds): *Excepta Medica APCS* **38**: 13–22

Moriyasu F, Gouchi A, Iijima T, Kouno R, Sunami M and Orita K (1985) Intratumoral administration of immune modulator PSK (Krestin) and its effect on lymphocyte population present in tumor and peripheral blood. In: *Recent Advances in Chemotherapy,* Ishigami J (ed) University of Tokyo Press: Tokyo pp 754–765

Nakazato H, Koike A, Saji S, Ogawa N and Sakamoto J (1994) Efficacy of immunochemotherapy as adjuvant treatment after curative resection of gastric cancer. *Lancet* **343**: 1122–1126

Niimoto M, Hattori T, Ito I, Tamada R, Inokuchi K, Orita K, Furue H, Ogawa N, Toda T and Furusawa M (1984) Levamisole in postoperative adjuvant immunochemotherapy for gastric cancer. A randomized controlled study of the MMC + tegafur regimen with or without levamisole Report 1. *Cancer Immunol Immunother* **18**: 13–18

Noguchi M and Miyazaki I (1996) Prognostic significance and surgical management of lymph node metastasis in gastric cancer. *Br J Surg* **83**: 156–161

Saito M, Ebina T, Koi M, Yamaguchi T, Kawada Y and Ishida N (1982) Induction of IFN-γ in mouse spleen cell by OK-432, a preparation of streptococcus pyogenes. *Cell Immunol* **68**: 187–192

Sato M, Harada K, Yoshida Y, Yura Y, Azuda M, lga H, Bendo T, Kousaata and Takegama (1997) Therapy for oral squamous cell carcinoma by tegafur and streptococcal agent OK-432 in combination with radiotherapy: Association of the therapeutic effect with differentiation and apoptosis in cancer cells. *Apoptosis* **2**: 227–238

Schenidel DJ, Gamsbacher B, Oberneder R, Kriegmair M, Hofstetter A, Riethmler G and Segrodo OG (1993) Tumor-specific lysis of human renal cell carcinomas by tumor infiltrating lymphocytes. *J Immunol* **151**: 4209–4220

Siewert JR, Bottcher K, Roder JD, Busch R, Hermanek P and Meyer HJ (1993) Prognostic relevance of systemic lymph node dissection in gastric carcinoma German Gastric Carcinoma Study Group. *Br J Surg* **80**: 1015–1018

Slovin SF, Lackman RD, Ferrone S, Kiely PE and Matstrangelo MJ(1986) Cellular immune response to human sarcoma; cytotoxic T cell clone reactive with autologous sarcomas. *J Immunol* **137**: 3042–3048

Stat Bite (1998) Stomach cancer mortality trends in U.S. men and women. *J Natl Cancer Inst* **90**: 185

Tanaka N, Gouchi A, Ohara T, Mannami T, Konaga E, Fuchimoto S, Okamura S, Sato K and Orita K(1994) Intratumoral injection of a streptococcal preparation, OK-432, before surgery for gastric cancer. A Randomized Trial. *Cancer* **74**: 3097–3103

The Japanese Gastric Cancer Study Group (1981) Gastric cancer registry in Japan – 5 year survival rate of cases between 1963 and 1966. *Jpn J Cancer Clin* **27**: 543–563

Toda K, Shiraishi T, Hirotsu T, Fukuyama K, Mineta T, Kawaguchi S and Tabuchi K (1996) The cytocidal activity of OK-432-activated mononuclear cells against human glioma cells is partly mediated through the Fas ligand/Fas system. *Jpn J Cancer Res* **87**: 972–976

Topalian SL, Soloman D and Rosenberg SA(1989) Tumor-specific cytolysis by lymphocytes infiltrating human melanomas. *J Immunol* **142**: 3714–3725

Uchida A and Mickshe M (1983) Lysis of fresh human tumor cells by autologous peripheral blood lymphocytes and pleural effusion lymphocytes activated by OK-432. *J Natl Cancer Inst* **71**: 673–680

*© 2001 Cancer Research Campaign*

Watanabe Y and Iwa T (1984) Clinical value of immunotherapy for lung cancer by the streptococcal preparation OK-432. *Cancer* **53**: 248–253
Yamamoto A, Nagamatsu M, Usami H, Sugawara Y, Watanabe N, Niitsu Y and Urushizaki (1986) Release of tumor necrosis factor (TNF) into mouse peritoneal fluids by OK-432, a streptococcal preparation. *Immunopharmacol* **11**: 79–86
Yang KD, Stone RM, Lee CS, Chao TY, Cheng SN and Shaio MF (1992) Effect of picibanil (OK-432) on neutrophil-mediated antitumor activity: implication of monocyte-derived neutrophil-activating factors. *Cancer Immunol Immunother* **35**: 277–282
Yasumura S, Weidman E, Hirabayashi H, Johnson JT, Herberman RB and Whiteside TL (1994) HLA restriction and T-cell-receptor V6 gene expression of cytotoxic T lymphocytes reactive with human squamous cell carcinoma of the head and neck. *Int J Cancer* **57**: 297–305

*© 2001 Cancer Research Campaign*

## References

[PDF_01629] Arinaga S., Karimine N., Takamuku K., Nanbara S., Inoue H., Abe R., Watanabe D., Matsuoka H., Ueo H., Akiyoshi T. (1992). A trial of adjuvant chemoimmunotherapy with mitomycin C and OK-432 for stage III gastric carcinoma.. J Surg Oncol.

[PDF_01633] BLACK M. M., OPLER S. R., SPEER F. D. (1956). Structural representations of tumor-host relationships in gastric carcinoma.. Surg Gynecol Obstet.

[PDF_01638] Frost P., Ng C. P., Belldegrun A., Bonavida B. (1997). Immunosensitization of prostate carcinoma cell lines for lymphocytes (CTL, TIL, LAK)-mediated apoptosis via the Fas-Fas-ligand pathway of cytotoxicity.. Cell Immunol.

[PDF_01642] Gotoh K., Gouchi A., Akura Y., Tanaka N., Orita K. (1991). Augmentation of cytotoxicity of tumor-infiltrating lymphocytes by biological response modifiers.. Int J Immunopharmacol.

[PDF_01648] Howson C. P., Hiyama T., Wynder E. L. (1986). The decline in gastric cancer: epidemiology of an unplanned triumph.. Epidemiol Rev.

[PDF_01653] Ikeda H., Sato N., Matsuura A., Sasaki A., Takahashi S., Kozutsumi D., Kobata T., Okumura K., Wada Y., Hirata K. (1996). Clonal dominance of human autologous cytotoxic T lymphocytes against gastric carcinoma: molecular stability of the CDR3 structure of the TCR alphabeta gene.. Int Immunol.

[PDF_01658] Inokuchi K., Hattori T., Taguchi T., Abe O., Ogawa N. (1984). Postoperative adjuvant chemotherapy for gastric carcinoma. Analysis of data on 1805 patients followed for 5 years.. Cancer.

[PDF_01661] Ioannides C. G., Fisk B., Jerome K. R., Irimura T., Wharton J. T., Finn O. J. (1993). Cytotoxic T cells from ovarian malignant tumors can recognize polymorphic epithelial mucin core peptides.. J Immunol.

[PDF_01664] Ishii Y., Yamaoka H., Toh K., Kikuchi K. (1976). Inhibition of tumor growth in vivo and in vitro by macrophages from rats treated with a streptococcal preparation, OK-432.. Gan.

[PDF_01667] Itoh K., Tilden A. B., Balch C. M. (1986). Interleukin 2 activation of cytotoxic T-lymphocytes infiltrating into human metastatic melanomas.. Cancer Res.

[PDF_01673] Kataoka K., Naomoto Y., Orita K. (1991). In vitro induction of perforin mRNA and cytotoxic activities in human splenocytes by the streptococcal preparation, OK-432.. Jpn J Clin Oncol.

[PDF_01670] Kägi D., Vignaux F., Ledermann B., Bürki K., Depraetere V., Nagata S., Hengartner H., Golstein P. (1994). Fas and perforin pathways as major mechanisms of T cell-mediated cytotoxicity.. Science.

[PDF_01679] Liu C. C., Walsh C. M., Young J. D. (1995). Perforin: structure and function.. Immunol Today.

[PDF_01681] Lopez C. B., Rao T. D., Feiner H., Shapiro R., Marks J. R., Frey A. B. (1998). Repression of interleukin-2 mRNA translation in primary human breast carcinoma tumor-infiltrating lymphocytes.. Cell Immunol.

[PDF_01684] Lowin B., Hahne M., Mattmann C., Tschopp J. (1994). Cytolytic T-cell cytotoxicity is mediated through perforin and Fas lytic pathways.. Nature.

[PDF_01690] Maehara Y., Moriguchi S., Sakaguchi Y., Emi Y., Kohnoe S., Tsujitani S., Sugimachi K. (1990). Adjuvant chemotherapy enhances long-term survival of patients with advanced gastric cancer following curative resection.. J Surg Oncol.

[PDF_01693] Maguire H. C., Ketcha K. A., Lattime E. C. (1997). Neutralizing anti-IL-10 antibody upregulates the induction and elicitation of contact hypersensitivity.. J Interferon Cytokine Res.

[PDF_01705] Nakazato H., Koike A., Saji S., Ogawa N., Sakamoto J. (1994). Efficacy of immunochemotherapy as adjuvant treatment after curative resection of gastric cancer. Study Group of Immunochemotherapy with PSK for Gastric Cancer.. Lancet.

[PDF_01708] Niimoto M., Hattori T., Ito I., Tamada R., Inokuchi K., Orita K., Furue H., Ogawa N., Toda T., Furusawa M. (1984). Levamisole in postoperative adjuvant immunochemotherapy for gastric cancer. A randomized controlled study of the MMC + Tegafur regimen with or without levamisole. Report I.. Cancer Immunol Immunother.

[PDF_01713] Noguchi M., Miyazaki I. (1996). Prognostic significance and surgical management of lymph node metastasis in gastric cancer.. Br J Surg.

[PDF_01715] Saito M., Ebina T., Koi M., Yamaguchi T., Kawade Y., Ishida N. (1982). Induction of interferon-gamma in mouse spleen cells by OK-432, a preparation of Streptococcus pyogenes.. Cell Immunol.

[PDF_01718] Sato M., Harada K., Yoshida H., Yura Y., Azuma M., Iga H., Bando T., Kawamata H., Takegawa Y. (1997). Therapy for oral squamous cell carcinoma by tegafur and streptococcal agent OK-432 in combination with radiotherapy: association of the therapeutic effect with differentiation and apoptosis in the cancer cells.. Apoptosis.

[PDF_01723] Schendel D. J., Gansbacher B., Oberneder R., Kriegmair M., Hofstetter A., Riethmüller G., Segurado O. G. (1993). Tumor-specific lysis of human renal cell carcinomas by tumor-infiltrating lymphocytes. I. HLA-A2-restricted recognition of autologous and allogeneic tumor lines.. J Immunol.

[PDF_01726] Siewert J. R., Böttcher K., Roder J. D., Busch R., Hermanek P., Meyer H. J. (1993). Prognostic relevance of systematic lymph node dissection in gastric carcinoma. German Gastric Carcinoma Study Group.. Br J Surg.

[PDF_01729] Slovin S. F., Lackman R. D., Ferrone S., Kiely P. E., Mastrangelo M. J. (1986). Cellular immune response to human sarcomas: cytotoxic T cell clones reactive with autologous sarcomas. I. Development, phenotype, and specificity.. J Immunol.

[PDF_01734] Tanaka N., Gouchi A., Ohara T., Mannami T., Konaga E., Fuchimoto S., Okamura S., Sato K., Orita K. (1994). Intratumoral injection of a streptococcal preparation, OK-432, before surgery for gastric cancer. A randomized trial. Cooperative Study Group of Preoperative Intratumoral Immunotherapy for Cancer.. Cancer.

[PDF_01741] Toda K., Shiraishi T., Hirotsu T., Fukuyama K., Mineta T., Kawaguchi S., Tabuchi K. (1996). The cytocidal activity of OK-432-activated mononuclear cells against human glioma cells is partly mediated through the Fas ligand/Fas system.. Jpn J Cancer Res.

[PDF_01745] Topalian S. L., Solomon D., Rosenberg S. A. (1989). Tumor-specific cytolysis by lymphocytes infiltrating human melanomas.. J Immunol.

[PDF_01747] Uchida A., Micksche M. (1983). Lysis of fresh human tumor cells by autologous peripheral blood lymphocytes and pleural effusion lymphocytes activated by OK432.. J Natl Cancer Inst.

[PDF_01750] Watanabe Y., Iwa T. (1984). Clinical value of immunotherapy for lung cancer by the streptococcal preparation OK-432.. Cancer.

[PDF_01752] Yamamoto A., Nagamuta M., Usami H., Sugawara Y., Watanabe N., Niitsu Y., Urushizaki I. (1986). Release of tumor necrosis factor (TNF) into mouse peritoneal fluids by OK-432, a streptococcal preparation.. Immunopharmacology.

[PDF_01755] Yang K. D., Stone R. M., Lee C. S., Chao T. Y., Cheng S. N., Shaio M. F. (1992). Effect of picibanil (OK432) on neutrophil-mediated antitumor activity: implication of monocyte-derived neutrophil-activating factors.. Cancer Immunol Immunother.

[PDF_01759] Yasumura S., Weidmann E., Hirabayashi H., Johnson J. T., Herberman R. B., Whiteside T. L. (1994). HLA restriction and T-cell-receptor V beta gene expression of cytotoxic T lymphocytes reactive with human squamous-cell carcinoma of the head and neck.. Int J Cancer.

